# Suicide Risk Associated with Experience of Violence and Impulsivity in Alcohol Dependent Patients

**DOI:** 10.1038/srep19373

**Published:** 2016-01-19

**Authors:** Lotfi Khemiri, Jussi Jokinen, Bo Runeson, Nitya Jayaram-Lindström

**Affiliations:** 1 Department of Clinical Neuroscience, Center for Psychiatry Research, Karolinska Institutet, Stockholm, Sweden; 2 Department of Clinical Sciences, Umeå University, Umeå, Sweden

## Abstract

Alcohol dependence (AD) and aggression-impulsivity are both associated with increased suicide risk. There is a need to evaluate clinical tools in order to improve suicide risk assessment of AD patients. The present study consisted of 95 individuals with a diagnosis of AD, consecutively admitted for addiction treatment, compared with 95 healthy controls. Suicidal risk was assessed together with exposure of violence and impulsivity. AD patients reported significantly higher rates of exposure to violence in childhood, as measured by the Karolinska Interpersonal Violence Scale (KIVS), compared to HC. Within the AD group, individuals with history of suicidal ideation and suicidal behavior reported higher levels of violence experience compared to AD individuals without such history. AD patients with previous suicidal ideation scored higher on self-reported impulsivity as assessed by the Barratt Impulsivity Scale (BIS). Our main finding was that experience of trauma and expression of violent behavior, coupled with increased impulsivity are associated with an elevated suicide risk in AD patients. Future longitudinal studies assessing these traits are needed to evaluate their potential role in identifying AD patients at risk of future suicide.

Alcohol dependence (AD) is associated with increased risk of suicide, and this has been demonstrated in several lines of research, including post-mortem studies of suicide cases^1^,^2^,^3^,^4^ and larger cohort studies^5^,^6^. The lifetime risk of suicide in AD patients has been estimated to 7%^6^, and co-morbid substance use disorder increases the absolute risk of suicide in all mental disorders^7^. Compared to the general population, individuals with a diagnosis of AD are almost 10 times more likely to die by suicide and those with drug dependence are 14 times more likely to commit suicide^8^. Suicidal behavior in AD patients has been associated with several risk factors such as severity of disease^9^,^10^, family history of AD^10^,^11^, family history of suicide attempts^9^,^12^, earlier onset of alcohol-related problems^10^,^11^ and psychiatric co-morbidity^9^,^10^,^11^,^12^,^13^. Although addiction treatment programs are sometimes reluctant to accept patients with recent suicidal behavior, up to 40% of patients seeking treatment for substance dependence report a history of suicide attempts^12^,^14^. Thus, suicidal behavior is a significant clinical problem among individuals in addiction treatment and it is important to identify risk factors and develop feasible clinical tools to characterize the AD patients at an elevated risk of future suicide.

The clinical assessment and categorization of suicide-related behaviors are inherently difficult and have been inconsistent across different clinical settings. The Columbia-Suicide Severity Rating Scale (C-SSRS) was developed to address previous inconsistencies, and comprises a clear distinction between suicidal ideation and suicidal behavior respectively^15^. The FDA recommends using the C-SSRS in clinical trials^16^, and suicide risk in the present study was operationalized as history of suicidal ideation and suicidal behavior, as assessed by this rating scale.

It is well known that exposure to violence in childhood^17^ and aggressive behaviors are known risk factors for suicidal behavior^18^,^19^. Also, higher level of aggression is associated with non-fatal suicide attempts^20^ and completed suicide^21^,^22^. Previous studies in AD individuals have shown that personality traits such as behavioral disinhibition and aggression are linked to suicidal behavior^23^,^24^. Conner *et al*. (2001) have found that violent behavior in the last year increases the risk of completed suicide in individuals with and without history of alcohol abuse. In a recent study, our co-author (JJ) and colleagues constructed and validated a brief 4-item rating instrument that quantifies exposure to, and expression of, violent acts in childhood and adult life, called the Karolinska Interpersonal Violence Scale (KIVS)^25^. In a prospective study of 161 recent suicide attempters, KIVS score predicted completed suicide at 4-year follow-up^25^. To our knowledge, the association between experience of violence measured by the KIVS and suicide risk has previously not been evaluated in treatment seeking AD patients.

Impulsivity, i.e. acting without foresight or an inability to inhibit prepotent responses^26^, is a personality trait proposed to mediate suicidal behavior. In suicide research, impulsive and aggressive behavior are often combined to one construct entitled “Aggression-Impulsivity”, and is strongly associated with suicidal behavior^18^,^21^,^22^,^27^. It is well established that AD patients have increased levels of impulsivity compared to healthy controls^28^,^29^, but only a few previous clinical studies have investigated the association between impulsivity and suicide risk in this patient population. In a theoretical model proposed by Conner and Duberstein^30^, aggression-impulsivity has been suggested as a key predisposing factor for suicide in AD patients. Wojnar *et al*.^31^ found that performance on a response inhibition task distinguished AD patients with a history of impulsive suicide attempt from those with a non-impulsive suicide attempt. Furthermore, AD patients with previous suicidal behavior scored higher on self-rated trait impulsivity^31^,^32^. In the current study we investigated whether impulsivity measured by the widely used Barratt Impulsivity Scale (BIS) is associated with different levels of suicide risk in AD patients.

Addiction treatment facilities may fail to identify patients at an elevated risk of suicide since the treatments primarily focus on substance use. In addition, commonly used suicide risk assessments such as the SAD PERSONS scale^33^ are aimed at capturing more traditional risk factors (e.g. previous suicide attempt) and do not include items regarding either violence experience or impulsivity. It is thus possible that clinical assessment of violence experience and impulsivity may be critical to identify a subgroup of AD patients at high risk of suicide. In the present study we therefore investigated the association between self-rated impulsivity, experience of violence and suicide risk in AD patients in an outpatient addiction treatment facility.

## Methods

### Participants

Ninety-five AD patients were recruited consecutively through public advertising and invited to the out patient treatment research clinic at the Stockholm Centre for Dependency Disorders to enroll in treatment studies. Participating AD patients provided informed consent and were explicitly informed that all data they provided would be summarized on group level and considered confidential. All patients underwent physical examination, performed a breathalyzer and urine dip test and were interviewed using the Structured Clinical Interview for DSM IV axis I disorders^34^ and Time-Line Follow-Back interview^35^ to assess drinking during the last 90 days. Heredity for AD and suicide in first or second-degree relatives were based on self-report. Inclusion criteria were: Male or female age 18–65, minimum nine years of education and currently fulfilling DSM-IV criteria for AD. Exclusion criteria were: Fulfills DSM-IV criteria for schizophrenia, bipolar disorder, major depression or any other substance dependence (excluding nicotine), previous withdrawal-induced delirium tremens or seizures, current severe somatic illness e.g. liver cirrhosis, use of any narcotics the last 30 days, positive urine dip test for any narcotic substance or positive breathalyzer on day of study participation.

Ninety-five healthy controls (HC) were recruited as part of a study at the Suicide Prevention Clinic at the Karolinska University Hospital, and data from these subjects have been reported previously^25^. All HC’s provided informed consent and were screened by a psychiatrist to exclude any current and past mental disorder, including suicidal behavior. The studies comprising the AD and HC populations were approved by the regional ethical review board in Stockholm, and conducted in accordance with the Declaration of Helsinki and Good Clinical Practice.

### Columbia-Suicide Severity Rating Scale (C-SSRS)

The C-SSRS is a semi-structured interview that evaluates suicidal ideation and suicidal behavior, respectively^15^,^36^. It consists of four constructs evaluating current and lifetime history of: (1) Severity of suicidal ideation rated on a 5-point ordinal scale where 1 = “wish to be dead”, 2 = “nonspecific active suicidal thoughts”, 3 = “suicidal thoughts with methods”, 4 = “suicidal intent”, and 5 = “suicidal intent with plan”; (2) Intensity of suicidal ideation comprised of 5 items: frequency, duration, controllability, deterrents and reasons for ideation. All items were rated on a 5-point scale where higher points indicates greater intensity; (3) Suicidal behavior rated categorically yes/no for actual attempt; aborted attempt; interrupted attempt; preparatory behavior (e.g. writing suicide letter) in contrast to non-suicidal self-injury behavior; (4) Lethality of actual suicide attempts. All C-SSRS interviews were performed by an M.D., and we report lifetime history of suicidal ideation severity and suicidal behavior.

### The Karolinska Interpersonal Violence Scale (KIVS)

The KIVS^25^ consists of 4 subscales assessing exposure to violence and expressed violent behavior in childhood (6–14 years old) and adulthood (15 years or older). Each item is scored from 0–5 where greater score indicates more severe experiences of violence. In the HC, the assessment was done as a semi-structured interview by trained clinicians. The AD patients completed the ratings through self-report. See Table 1 for the complete KIVS items and scoring. The KIVS has previously been shown to have high inter-rater reliability as well as validity^25^ and has been used in several suicide research studies^37^,^38^.

### Barratt Impulsivity Scale (BIS)

The Barratt Impulsivity Scale (BIS) was designed to measure the personality construct of impulsivity and is currently in it’s 11^th^ revised form^39^. It consists of 30 self-report-items (e.g. “I say things without thinking”) each rated using a 4-point ordinal scale where 1 = “Rarely/never”, 2 = “Occasionally”, 3 = “Often” and 4 = “Almost always/always”. Outcomes reported in this study are the total BIS score, as well as the three subscales of attentional impulsivity, motor impulsivity and non-planning impulsivity. The English version of the BIS was translated to Swedish and back-translated by an authorized bilingual translator.

### Statistical Analysis

Outcomes are reported using the mean, median, standard deviation and range. The assumption of normality was evaluated using the Shapiro-Wilks test. Between-group comparisons were done using two-tailed students t-test and Fischer’s exact test for continuous and categorical variables, respectively. If data were not normally distributed non-parametric tests were used.

We performed step-wise comparisons of KIVS and BIS (total score and all the subscales) between the following groups: (1) AD patients and HC; (2) AD patients with and without previous suicidal ideation; (3) AD patients with and without previous suicidal behavior; (4) AD patients with and without previous suicide attempt. In order to investigate potential confounding factors, these subgroups of AD patients were also compared regarding clinical characteristics (sex, age) as well as known risk factors for suicidal behavior in AD patients (previous depression, alcohol consumption, dependence severity, heredity for AD and heredity for suicide). Since the KIVS scores were positively skewed, all comparisons between groups were performed using the non-parametric Mann-Whitney U test. The BIS however was normally distributed, and students t-tests were performed to compare groups. Spearman correlation coefficients were calculated to assess correlations between KIVS subscales, as well as KIVS and BIS score. Statistical analyses were performed using SPSS version 21. No missing data was imputed, and analyses include only those subjects who completed all items on each questionnaire or subscale. The alpha level was set to 0.05, uncorrected, two-tailed.

## Results

### Participants

The AD patients had a mean age of 47 years (SD = 7.3; range 24–58) and 43% (n = 41) were female. The average number of fulfilled DSM IV criteria for AD was 5.2 (SD = 1.2; range 3–7) and 19% (n = 18) had a previous episode of major depressive disorder. In the AD group, 39% (n = 37) and 9.5% (n = 9) had a history of previous suicidal ideation and suicidal behavior, respectively. Among the 9 subjects with previous suicidal behavior, 4 had made previous suicide attempts while the other 5 had a history of aborted attempt and/or preparatory suicidal behavior. In the AD group, 2 subjects did not complete the C-SSRS and they were thus only included in analysis comparing HC and AD regarding KIVS score. In the HC the mean age was 40.2 years (SD = 11; range 18–63) and 60% were female (n = 57). The HC group was younger than the AD group (t(187) = −5.1; p < 0.001) and had more females (p = 0.030). Table 2 provides a summary of experience of violence and impulsivity measures across different levels of suicide risk.

### Experience of violence: Comparison of AD Patients and HC

Since the KIVS scores deviated from the normal assumption (all Shapiro-Wilks tests p < 0.001), comparisons were performed using non-parametric tests. There was a trend toward greater overall violence experience (KIVS total score) in the AD group (n = 95) compared to the HC (n = 95; Z = 1.9; p = 0.052). The rating of exposure to violence during childhood was significantly higher in the AD group (Z = 2.6; p = 0.009) while there were no significant differences regarding the other three subscales (all p > 0.1; Table 3). The different KIVS subscales of exposure to and expression of violence were all significantly correlated in the entire sample (Table 4). Within the AD group, males rated significantly higher regarding expression of violent behavior during childhood compared to females (Z = −3.7; p < 0.001). There were no gender differences in any of the other KIVS subscales (all p > 0.05). Within the HC group, there were no gender differences in any of the KIVS subscales (all p > 0.05).

### Experience of violence: Comparison of AD patients with and without history of suicidal ideation

**Figure 1:**
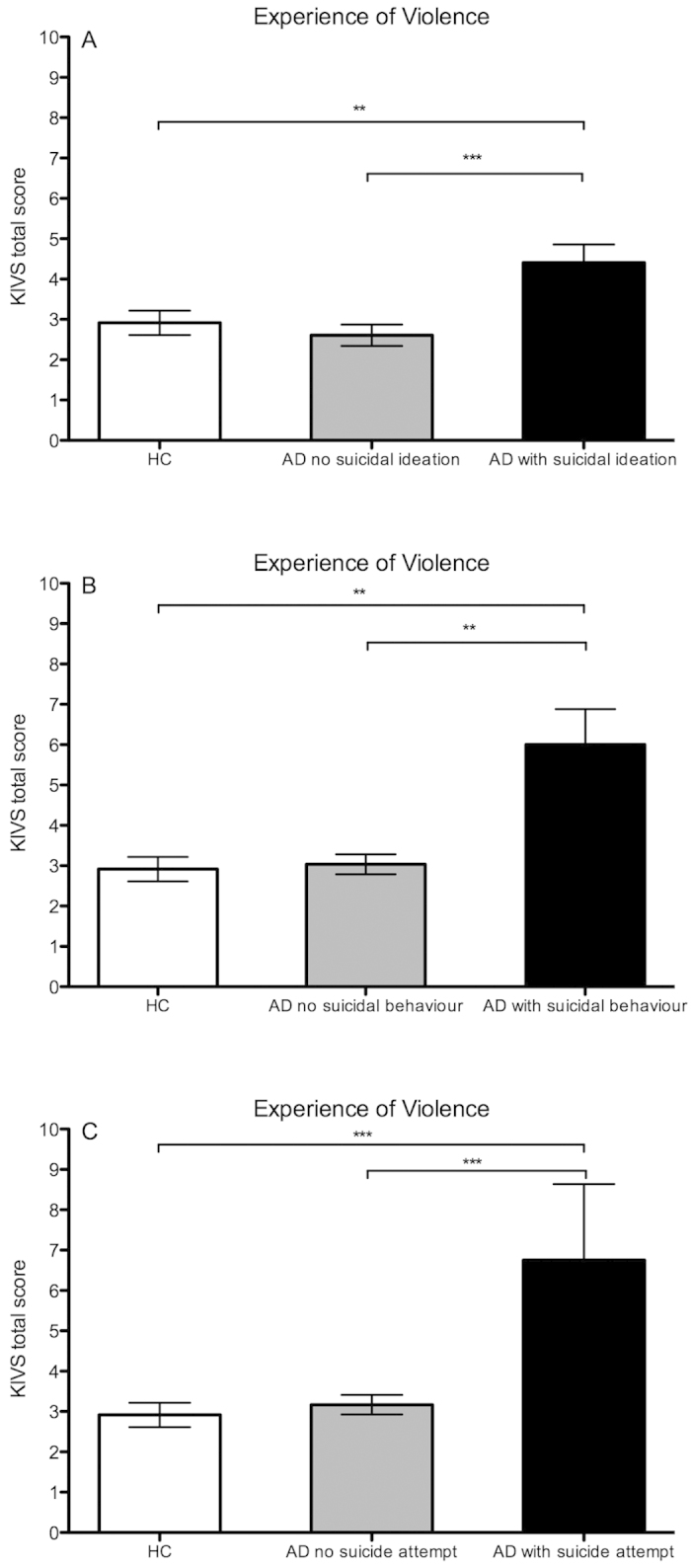
Karolinska Interpersonal Violence Scale (KIVS) ratings of experience of violence in alcohol dependent (AD) patients with increasing suicide risk as assessed by the Columbia-Suicide Severity Rating Scale (C-SSRS). (**A**) HC (n = 95) versus AD patients without suicidal ideation (n = 58) versus AD patients with suicidal ideation (n = 37); (**B**) HC versus AD patients without suicidal behavior (n = 86) versus AD patients with suicidal behavior (n = 9); (**C**) HC versus AD patients without suicide attempt (n = 91) versus AD patients with suicide attempt (n = 4). *p < 0.05; **p < 0.01; ***p < 0.001.

**Figure 2:**
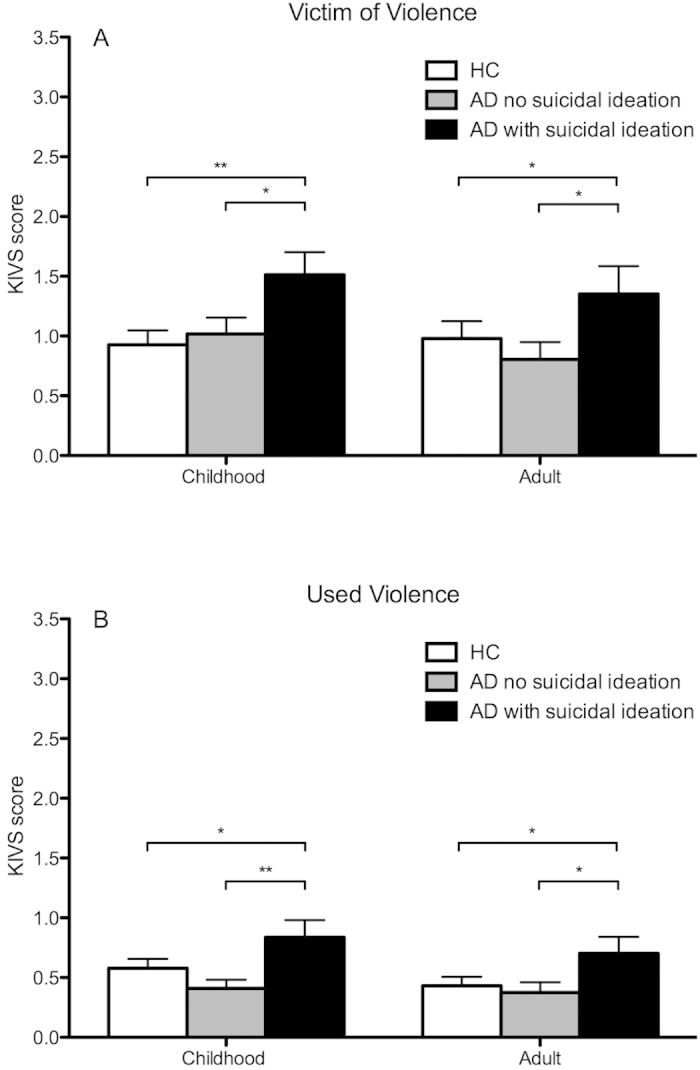
Karolinska Interpersonal Violence Scale (KIVS) subscale scores measuring exposure to (**A**) and expression of (**B**) violent acts in childhood and adulthood respectively, in alcohol dependent patients (AD) with (n = 37) and without (n = 58) history of suicidal ideation and healthy controls (HC; n = 95). *p < 0.05; **p < 0.01; ***p < 0.001.

AD patients with a history of previous suicidal ideation (1 or more on the C-SSRS lifetime suicidal ideation construct; n = 37) reported significantly greater violence experience (Z = 3.6; p < 0.001; Fig. 1A) as well as all four subscales (all p < 0.05; Fig. 2) compared to AD patients without such history (n = 58). Within the AD group with previous suicidal ideation, there was a significant correlation between severity of suicidal ideation and KIVS total score (r = 0.40; p = 0.01). Among the subscales, there was a significant correlation between severity of suicidal ideation and expression of violence as adult (r = 0.33; p = 0.048), and no significant correlations for any of the three other subscales (all p > 0.1).

There was no difference in overall violence experience between HC and AD patients without previous suicidal ideation (Z = −0.05; p = 0.96), while AD patients with previous suicidal ideation scored significantly higher than HC on total KIVS score (Fig. 1A) as well as all the subscales (Fig. 2).

There was no statistically significant difference between AD patients with and without history of suicidal ideation regarding number of drinks consumed the last 90 days (t(88) = 0.63; p = 0.53), age (t(90) = 1.0; p = 0.32), sex (p = 0.67) heredity for AD (p = 0.17) or heredity for suicide (p = 0.75). The AD patients with previous suicidal ideation fulfilled more DSM-IV AD criteria (mean 5.5 vs 5.0;t(88) = −2.1; p = 0.038) and had to a higher degree a previous depressive episode (35% vs 9%; p = 0.030) compared with those without previous suicidal ideation.

### Experience of violence: Comparison of AD patients with and without history of suicidal behavior

**Figure 3:**
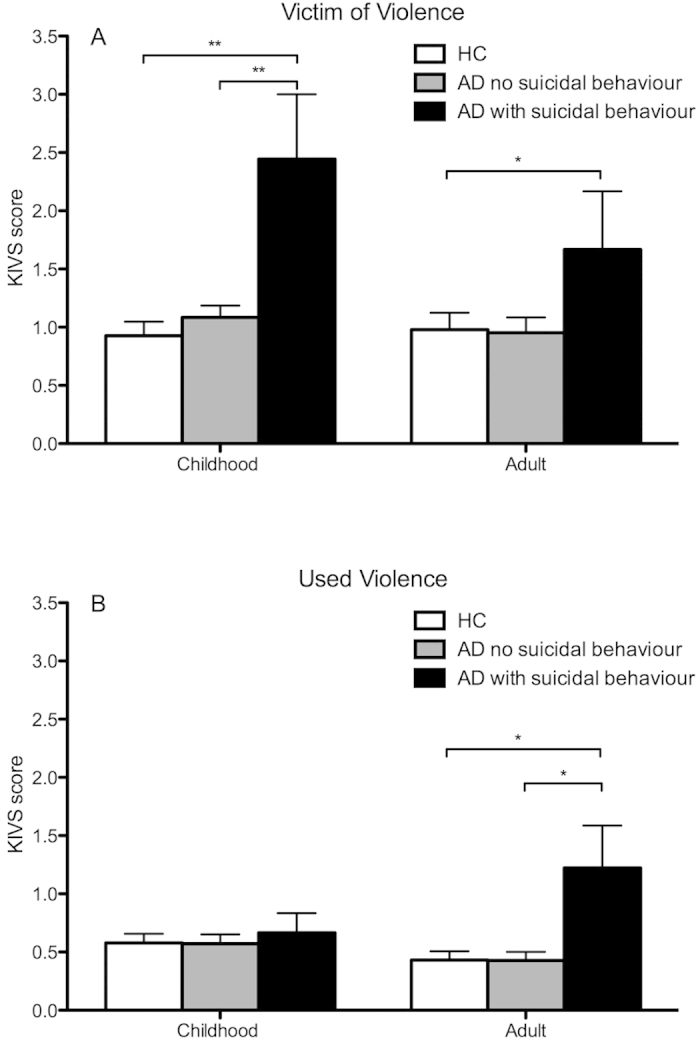
Karolinska Interpersonal Violence Scale (KIVS) subscale scores measuring exposure to (**A**) and expression of (**B**) violent acts in childhood and adulthood respectively, in alcohol dependent patients (AD) with (n = 9) and without (n = 86) history of suicidal behavior and healthy controls (HC). *p < 0.05; **p < 0.01; ***p < 0.001.

AD patients with a history of suicidal behavior (n = 9; responding yes to any item on the C-SSRS suicidal behavior construct excluding non-suicidal self-injury) reported significantly greater overall violence experience (Z = 3.4; p < 0.001; Fig. 1B), including subscales of expressed violence as adult (Z = 2.5; p = 0.014; Fig. 3B) and exposure to violence in childhood (Z = 2.7; p = 0.006; Fig. 3A), compared to AD patients without such history (n = 86).

There was no difference in overall violence experience between HC and AD patients without previous suicidal behavior (Z = −1.1; p = 0.27), while the AD patients with previous suicidal behavior scored significantly higher than HC regarding overall violence experience (Fig. 1B), including all the subscales except using violence in childhood (Fig. 3).

There was no statistically significant difference between AD patients with and without history of suicidal behavior regarding number of DSM-IV AD criteria (t(88) = 0.41; p = 0.69), drinks consumed the last 90 days (t(88) = 0.67; p = 0.50, age (t(90) = 0.23; p = 0.82), sex (p = 1.0), heredity for AD (p = 1.0) or heredity for suicide (p = 1.0). The AD patients with previous suicidal behavior had to a higher degree a previous depressive episode (44% vs 17%; p = 0.072) compared to those without previous suicidal behavior.

### Experience of violence: Comparison of AD patients with and without history of suicide attempt

Only four patients had a history of previous suicide attempt, and this group reported significantly greater overall violence experience compared to AD patients without any previous suicide attempt (Z = 2.3; p = 0.021) as well as the HC group (Z = 2.3; p = 0.019; Fig. 1C). No statistically significant difference in any of the subscales was observed (data not shown; p > 0.1 for all subscales).

### Violence Experience: Sensitivity Analyses

Since the HC were significantly younger and to a higher degree female, compared to AD patients, the following sensitivity analysis was performed: The youngest females in the HC group (n = 15) and the oldest males in the AD group (n = 15) were removed from analysis. After removal of these subjects, there was no significant difference between the remaining AD and HC regarding age (HC: 43.5; AD: 45.7; t(156) = −1.7; p = 0.085) or sex (p = 0.94). Analysis of KIVS score yielded similar results as the original analysis. The AD patients scored significantly higher on KIVS total score (Z = 2.0; p = 0.046) and on the rating of exposure to violence during childhood (Z = 2.9; p = 0.004) while no significant difference was found for the other three subscales (all p > 0.1).

Since history of depressive episodes was more frequent in subjects with previous suicidal ideation and behavior, an additional sensitivity analysis was done. After excluding all subjects with history of major depression, the remaining subjects with history of suicidal ideation (Z = 3.1; p = 0.001) as well as behavior (Z = 2.4; p = 0.014) reported significantly greater overall violence experience compared to subjects without such history.

Since all AD patients with previous suicidal behavior also scored positive regarding previous suicidal ideation, an additional analysis was done to investigate whether these subjects alone drove the observed association. All AD patients were subdivided into the following three groups based on their most severe level of suicidality: 1) No previous suicidal ideation or behavior (n = 56; KIVS total score = 2.6); 2) Previous suicidal ideation but no suicidal behavior (n = 28; KIVS total score = 3.9) and 3) Previous suicidal ideation and previous suicidal behavior (n = 9; KIVS total score = 6.0). In line with the original analysis, both group 3(p < 0.001) and group 2(p = 0.014) reported significantly greater violence experience compared to both group 1.

### Impulsivity and suicide risk in AD patients

A subset of AD patients completed the Barratt Impulsivity Scale (BIS). AD patients with a history of suicidal ideation (n = 28) reported significantly higher overall impulsivity (BIS total score; mean: 66.1 vs 59.9; t(73) = 2.5; p = 0.014) compared to AD patients without such history (n = 47). Among the BIS subscales, the AD patients with previous suicidal ideation scored significantly higher on non-planning impulsivity (mean 27.0 vs 24.3; t(75) = 2.0; p = 0.046) and a trend was observed for motor impulsivity (22.8 vs 21.2; t(74) = 1.8; p = 0.071), but no difference in attentional impulsivity (mean 15.8 vs 14.6; t(76) = 1.4; p = 0.16). However, when comparing AD patients with (n = 8) and without previous suicidal behavior (n = 67) we found no significant difference regarding overall impulsivity (mean 61.1 vs 62.3; t(73) = −0.29; p = 0.77) or any of the subscales measuring non-planning (mean 24.9 vs 25.4; t(75) = −0.25; p = 0.80), motor (mean 22.1 vs 21.8; t(74) = 0.23; p = 0.82) and attentional impulsivity (mean 14.1 vs 15.2; t(76) = −0.82; p = 0.41). No difference was found between AD patients with (n = 4) and without (n = 71) previous suicide attempt (data not shown; All p > 0.1 for total BIS score and all subscales).

There were no statistically significant correlations between BIS and KIVS total score, nor the different KIVS subscales measuring exposure to and expression of violence (data not shown; All p > 0.1).

## Discussion

The main finding of the present study was that AD patients with a history of suicidal ideation and suicidal behavior, report increased levels of experience of violence. We also found that suicidal ideation, but not behavior, was associated with self-rated impulsivity. Collectively our findings indicate that trauma, traits of aggression and impulsivity might be critical factors contributing to the pathogenesis of suicidal behavior in the AD population.

Our findings are corroborated by several previous studies that have shown an association between both violence/aggression and suicide in general among individuals with mental disorders^18^,^20^,^21^,^22^,^27^, and specifically in AD patients^30^,^40^. In the study by Jokinen *et al*.^25^ it was shown that experience of violence as measured by the KIVS, specifically the subscales of victimization of violence in childhood and expression of violence in adulthood, predicted completed suicide in suicide attempters. In the present study AD patients with highest suicide risk, i.e. previous suicidal behavior, scored significantly higher, specifically on the same subscales that have previously been shown to predict completed suicide^25^. In addition, our results indicate that an overall experience of violence was increased in AD patients with incremental levels of suicide risk. Furthermore, the correlation between suicidal ideation severity and overall violence experience was statistically significant. Taken together these findings indicate that there may be a dose-response relationship between experience of violence and suicide risk in AD patients. The finding collectively provides valuable clinical evidence that in AD patients receiving outpatient addiction care, it is possible to identify individuals at an elevated risk of suicide related behaviors by utilizing the KIVS. It is known that suicide completers do not always communicate their suicidal thoughts and sometimes have no previous suicidal behavior^41^. It is thus possible that a clinical tool assessing violence experience is able to more accurately identify patients at an elevated risk of suicide who otherwise could be considered as “low-risk” based on traditional suicide risk assessments.

Exposure to violence during childhood was not only elevated in AD patients with increased suicide risk, but it was also the only subscale that differentiated AD patients from HC. It is known that childhood maltreatment, such as physical, emotional and sexual abuse, increases the risk of developing AD^42^,^43^. One possible interpretation of our result is that childhood trauma increases risk of developing AD later in life, and that AD itself could act as an independent risk factor for suicide. However, experience of childhood adversities is a known independent risk factor mediating all forms of psychopathology^42^ as well as suicide attempts^44^. Furthermore, in a study of 196 AD patients Huang *et al*.^45^ found that childhood trauma increased risk of both psychiatric disorders as well as suicide attempts. Thus, it is more likely that experience of childhood trauma acts as a multiple risk factor increasing the risk of AD, other mental disorders as well as suicidal behavior. This illustrates the importance of assessing trauma during childhood, when performing clinical suicide risk assessments in AD patients, and our study suggests that the KIVS is a feasible clinical tool for this purpose.

The neurobiological correlate of our finding is not known, but there is a vast amount of literature indicating an association between serotonergic neurotransmission and both violence and suicide^46^,^47^. Recently it was shown that the KIVS subscale measuring experience of violence in childhood was associated with low levels of the serotonin metabolite 5-hydroxyindoleacetic acid (5-HIAA) in cerebrospinal fluid (CSF) in female suicide attempters^48^. Furthermore, emotional neglect was found to be associated with reduced central serotonergic neurotransmission as measured by a neuroendocrine challenge test in male AD patients^49^. It is possible that the underlying neurobiological correlate of the association between suicide risk and exposure to childhood trauma observed in the current study is also related to changes in serotonergic neurotransmission. Future studies combining clinical suicide risk assessments with biological samples are needed to investigate to understand the possible pathophysiological mechanism common to AD and suicide.

In the present study, we found significantly elevated levels of self-rated impulsivity in AD patients with previous suicidal ideation, but not in those with previous suicidal behavior or suicide attempt. There are several plausible explanations for this discrepancy. First of all, the variance in self-rated impulsivity was large (range 44–89; mean 62 ± 11) and our limited sample of individuals with suicidal behavior was likely insufficient to detect a difference. Secondly, self-rated impulsivity as a construct may be too heterogeneous to be specific for suicidal behavior. This is substantiated by recent findings that demonstrated that a behavioral measure of impulsivity compared to the self-rated measure more accurately distinguished AD patients with impulsive suicide attempts from those without^31^. We did not find any correlation between KIVS total score or any of the subscales and self-rated impulsivity, suggesting that these two constructs are separate and could possibly constitute two independent factors important in suicide risk assessment in AD patients.

In order to rule out potential confounding factors that could otherwise explain our findings, we compared clinical characteristics of AD patients with different levels of suicide risk. We found no significant difference between AD patients on the different levels of suicide risk regarding gender, age, alcohol consumption over the last 90 days or heredity for AD and suicide respectively, indicating that these factors did not drive the observed association. However we found that AD patients with previous suicidal ideation fulfilled more DSM-IV AD criteria’s, indicating that the observed association between violence experience and suicide risk could be mediated by severity of diagnosis of AD. With respect to past psychiatric diagnosis, we found that AD patients with previous suicidal ideation and behavior were more likely to have had a past history of a depressive episode. Schwandt *et al*.^50^ showed that childhood trauma influenced severity of dependence in a clinical sample of 280 AD patients, and that this association was mediated through neuroticism. In a large retrospective cohort study of 17337 subjects Dube *et al*.^44^ found that childhood abuse increased risk of future suicide attempt, and this relationship was partially mediated through both self-reported AD and depression. Our results are in line with previous studies, suggesting that the association between violence experience and suicide risk in AD patients is in part mediated through dependence severity and depressive affect.

There are some important limitations in our study that need to be addressed. First of all, the study sample was of limited size and consisted of cross-sectional data. We emphasize that our findings should be interpreted with caution until replicated, given the limited number of subjects with actual suicidal behavior (n = 9) and suicide attempts (n = 4). Secondly, we did not clinically assess personality disorders in the AD sample. It is possible that e.g. antisocial personality disorder, which is associated with violence^51^, impulsivity^26^ and alcohol use disorders^52^, could confound some of the observed associations. However a recent study in AD patients with history of suicide attempts reported no association with personality disorders with impulsive features such as borderline personality disorder or anti social personality disorder^32^. Moreover in the current sample of AD patients there was no previous psychiatric diagnosis or treatment of any personality disorder, indicating that unmeasured personality disorders likely did not explain the observed association. Thirdly, the HC and AD group were not matched regarding sex and age. A sensitivity analysis was done which yielded almost identical results as the original analysis, indicating that the mismatched groups did not bias the results. Lastly, in the HC group the KIVS was administered as a semi-structured interview by a trained clinician, while the AD group completed the KIVS rating through self-report, which possibly could bias the result. However, in a study of substance use disorder patients there were convergent correlations between interview and self-report regarding different forms of childhood trauma^53^. This suggests that the discrepancy in method of administration of the KIVS should not constitute a major bias in the present study.

In summary, AD patients with increased suicide risk have more experience of violence compared to AD patients with low suicide risk as well as HC. Furthermore, previous suicidal ideation in AD patients was associated with increased self-rated impulsivity. Future studies employing a prospective design are needed to investigate whether clinical assessment of violence experience and impulsivity can predict attempted and completed suicide in AD patients and other substance use disorders.

## Additional Information

**How to cite this article**: Khemiri, L. *et al*. Suicide Risk Associated with Experience of Violence and Impulsivity in Alcohol Dependent Patients. *Sci. Rep*. **6**, 19373; doi: 10.1038/srep19373 (2016).

## Figures and Tables

**Table 1:** The Karolinska Interpersonal Violence Scale (KIVS). Copyright 2010, Jussi Jokinen MD, PhD.

**The Karolinska Interpersonal Violence Scale**
The steps of this scale are defined by short statements about violent behavior. Based on an interview with the subject; use the highest score where one or more of the statements apply.
**A. Used violence.**
**As a child (6–14 years)**
**0** No violence.
**1** Occasional fights, but no cause for alarm among grown-ups in school or in the family.
**2** Fighter. Been in fights a lot.
**3** Often started fights. Hit a comrade who had been bullied. Continued hitting when the other had surrendered.
**4** Initiated bullying. Often hit other children, with fist or object.
**5** Caused serious physical injury. Violent toward adult(s). Violent behavior that led to intervention by social welfare authorities.
**As an adult (15 years or older)**
**0** No violence.
**1** Slapped or spanked children on occasion. Shoved or shook partner or another adult.
**2** Occasionally smacked partner or child. Fought when drunk.
**3** Assaulted partner drunk or sober. Repeated corporal punishment of child. Frequent fighting when drunk. Hit someone when sober.
**4** Instance of violent sexual abuse. Repeated battering/physical abuse of child or partner. Assaulted/attacked other persons frequently, drunk or sober.
**5** Killed or caused severe bodily harm. Repeated instances of violent sexual abuse. Convicted of crime of violence.
**B. Victim of violence.**
**Childhood (6 – 14 years)**
**0** No violence.
**1** Occasional slaps. Fights in school, of no great significance.
**2** Bullied occasionally for short period(s). Occasionally exposed to corporal punishment.
**3** Often bullied. Frequently exposed to corporal punishment. Beaten by drunken parent.
**4** Bullied throughout childhood. Battered/beaten up by schoolmates. Regularly beaten by parent or another adult. Beaten with objects. Sexually abused.
**5** Repeated exposure to violence at home or in school that resulted at least once in serious bodily harm. Repeated sexual abuse, or sexual abuse that resulted in bodily harm.
**Adulthood (15 years or older)**
**0** No violence.
**1** Threatened or subjected to a low level of violence on at least one occasion.
**2** Beaten by partner on occasion. Victim of purse snatching. Threatened with object.
**3** Threatened with a weapon. Robbed. Beaten by someone other than partner. Frequently beaten by partner.
**4** Raped. Battered.
**5** Repeatedly raped. Repeatedly battered. Severely battered, resulting in serious bodily harm.

**Table 2:** Demographics of alcohol dependent patients (N = 95) and healthy controls (N = 95).

	Alcohol Dependence (N = 95)	Alcohol Dependence with previous suicidal ideation (N = 37)	Alcohol Dependence with previous suicidal behavior (N = 9)	Alcohol Dependence with previous suicide attempt (N = 4)	Healthy Controls (N = 95)
KIVS					
Experience of violence (KIVS total score)	3.4 (2.5)	4.4(2.7)	6.0(2.6)	6.8(3.8)	2.9 (3.0)
Expressed violent behavior during childhood	0.60(0.72)	0.84(0.87)	0.67(0.50)	1.0(0.0)	0.59(0.77)
Expressed violent behavior as adult	0.52(0.76)	0.70(0.85)	1.2(1.1)	1.5(1.3)	0.43(0.74)
Exposure to violence during childhood	1.2(1.1)	1.5(1.1)	2.4(1.7)	2.5(2.1)	0.93(1.2)
Exposure to violence as adult	1.1(1.3)	1.4(1.4)	1.7(1.5)	1.8(2.2)	0.98(1.4)
BIS					
Impulsivity (BIS total score)	62.3(10.7)	66.1(11.6)	61.1(10.6)	54.5(7.5)	*
Attentional Impulsivity	15.1(3.5)	15.8(3.3)	14.1(2.0)	13.3(1.7)	*
Motor Impulsivity	21.8(3.8)	22.8(4.4)	22.1(5.0)	19.0(2.2)	*
Non-planning Impulsivity	25.4(5.6)	27.0(6.3)	24.9(4.8)	22.3(4.9)	*

Experience of violence was measured by the Karolinska Interpersonal Violence Scale (KIVS) and impulsivity was measured using the Barratt Impulsivity Scale (BIS). Values are presented as mean (standard deviations).

*Prevalence of previous suicidal ideation and BIS were not collected in healthy controls.

**Table 3:** Karolinska Interpersonal Violence Scale (KIVS) ratings of experience of violence in alcohol dependent patients (n = 95) and healthy controls (n = 95).

Rating	Alcohol Dependence				Healthy Controls				Statistic
	Mean	Median	SD	Range	Mean	Median	SD	Range	
Experience of violence (KIVS total score)	3.41	3	2.52	0–14	2.92	2	2.96	0–17	Z = 1.9 p = 0.052
Expressed violent behavior during childhood (6–14 years old)	0.60	1	0.72	0–5	0.58	0	0.77	0–4	Z = 0.53 p = 0.60
Expressed violent behavior as adult ( >15 years old)	0.52	0	0.76	0–3	0.43	0	0.74	0–3	Z = 1.0 p = 0.30
Exposure to violence during childhood (6–14 years old)	1.23	1	1.09	0–5	0.93	0	1.19	0–5	Z = 2.6 p = 0.009
Exposure to violence as adult (>15 years old)	1.06	1	1.27	0–5	0.98	0	1.41	0–5	Z = 1.2 p = 0.23

**Table 4:** Correlations between the different subscales of Karolinska Interpersonal Violence Scale (KIVS), measuring exposure to and expression of violent acts in childhood and adulthood, in the entire study sample (n = 190).

	Exposure to violence during childhood	Expressed violent behavior as adult	Exposure to violence as adult	Expressed violence total
Expressed violent behavior during childhood	0.237**	0.286***	0.258***	
Exposure to violence during childhood		0.243**	0.262***	
Exposure to violence total				0.386***

*p < 0.05; **p < 0.01; ***p < 0.001.
